# Supplementary material for: Mining the Biosynthetic Potential for Specialized Metabolism of a Streptomyces Soil Community
Source: Antibiotics (Basel). 2020 May 23;9(5):271. doi: 10.3390/antibiotics9050271 (PMC7277575; doi:10.3390/antibiotics9050271)
Supplement: Supplementary file 1 [file antibiotics-09-00271-s001.pdf]

Supplementary data

Mining the biosynthetic potential for specialized metabolism of a *Streptomyces* soil community

Matthieu NICAULT[1,2], Abdoul-Razak TIDJANI[1], Anthony GAUTHIER[1], Stéphane DUMARCAY[3], Eric GELHAYE[2], Cyril BONTEMPS[1], Pierre LEBLOND[1]

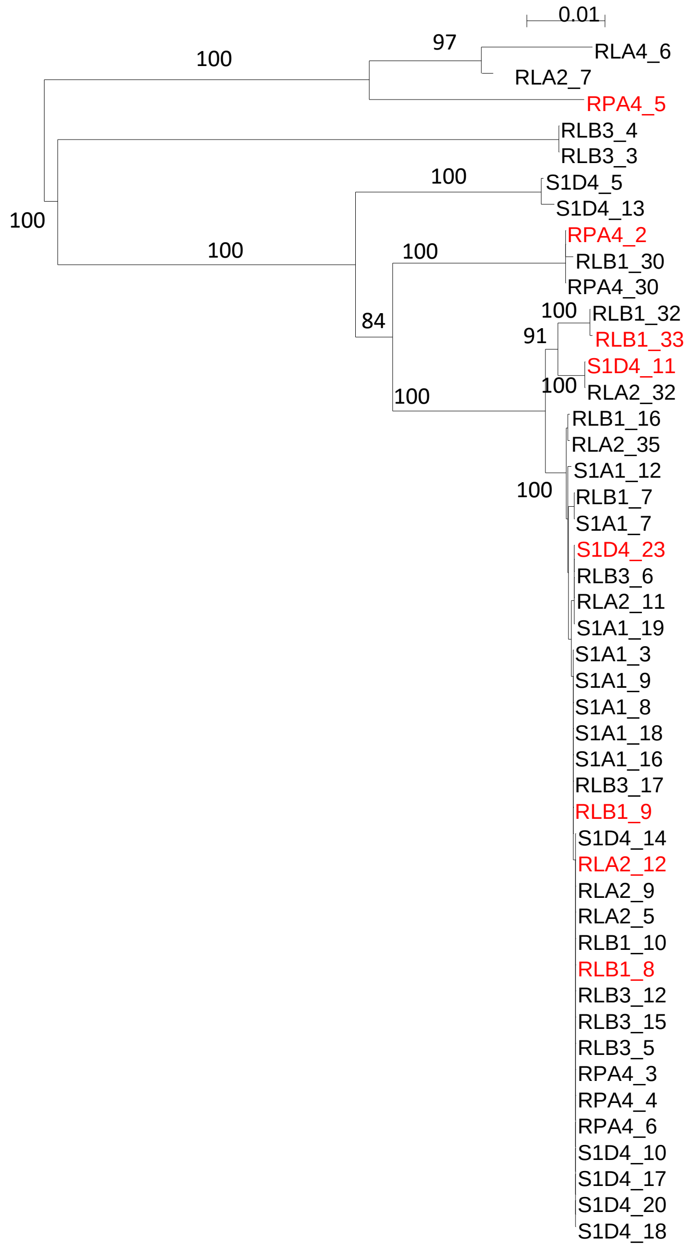

Fig. S1. Phylogenetic multi locus sequence typing (MLSA) tree of isolated strains.

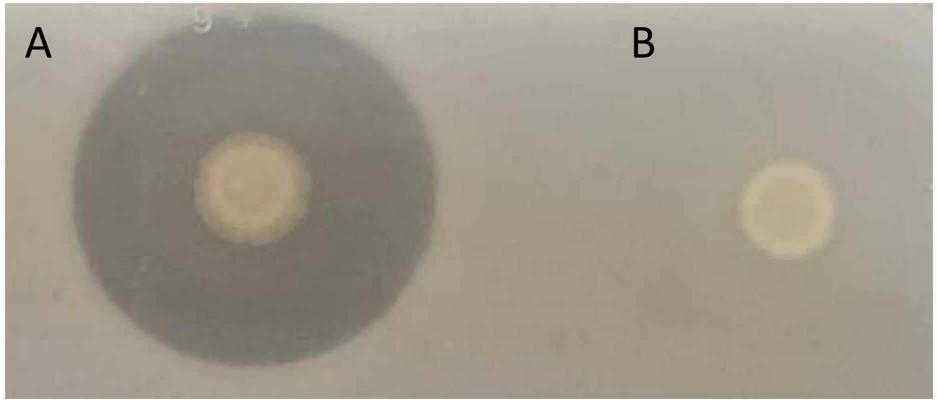

Fig. S2. Illustration of a bioassay test.

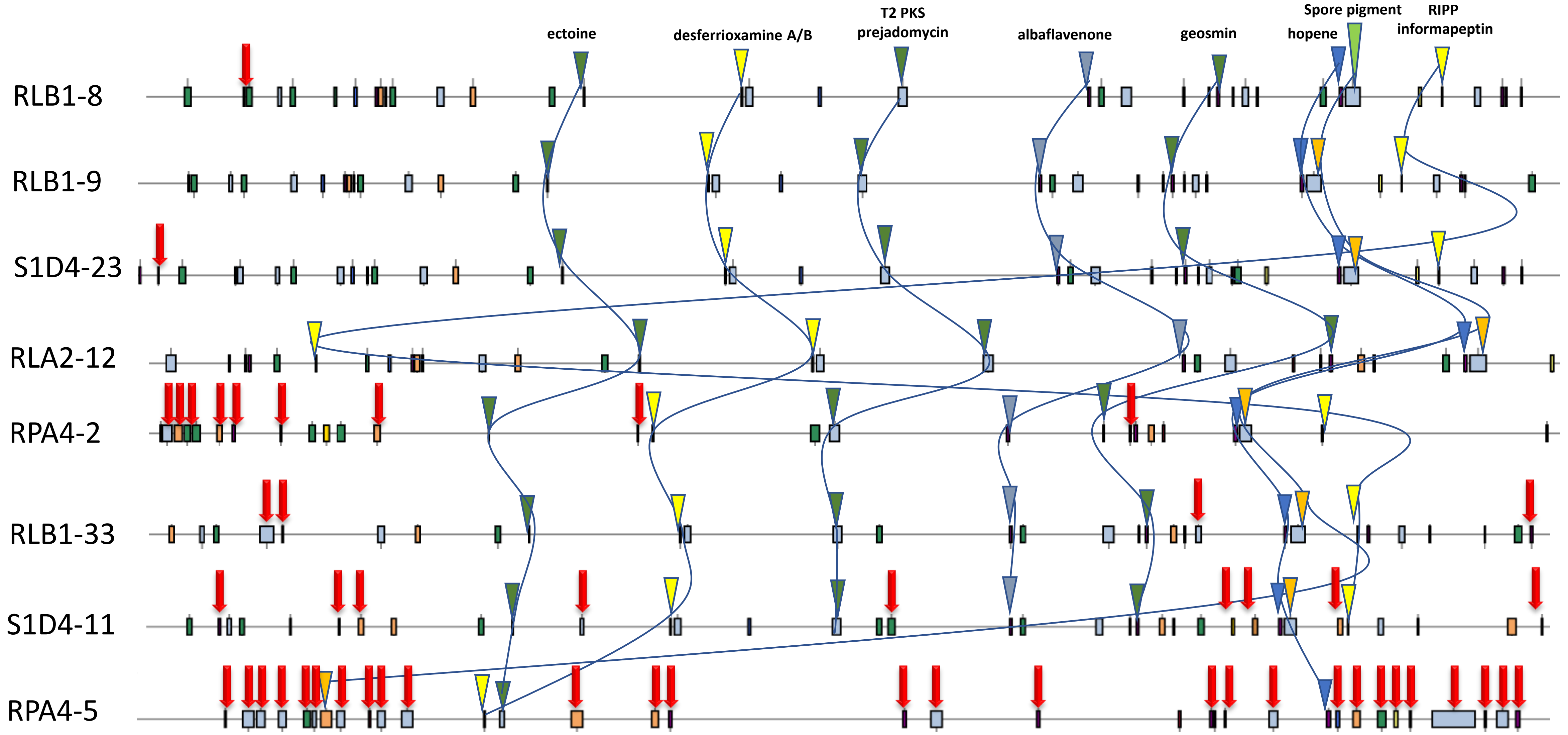

Fig S3. Schematization of the biosynthesis pathways along the chromosome of the strains studied.

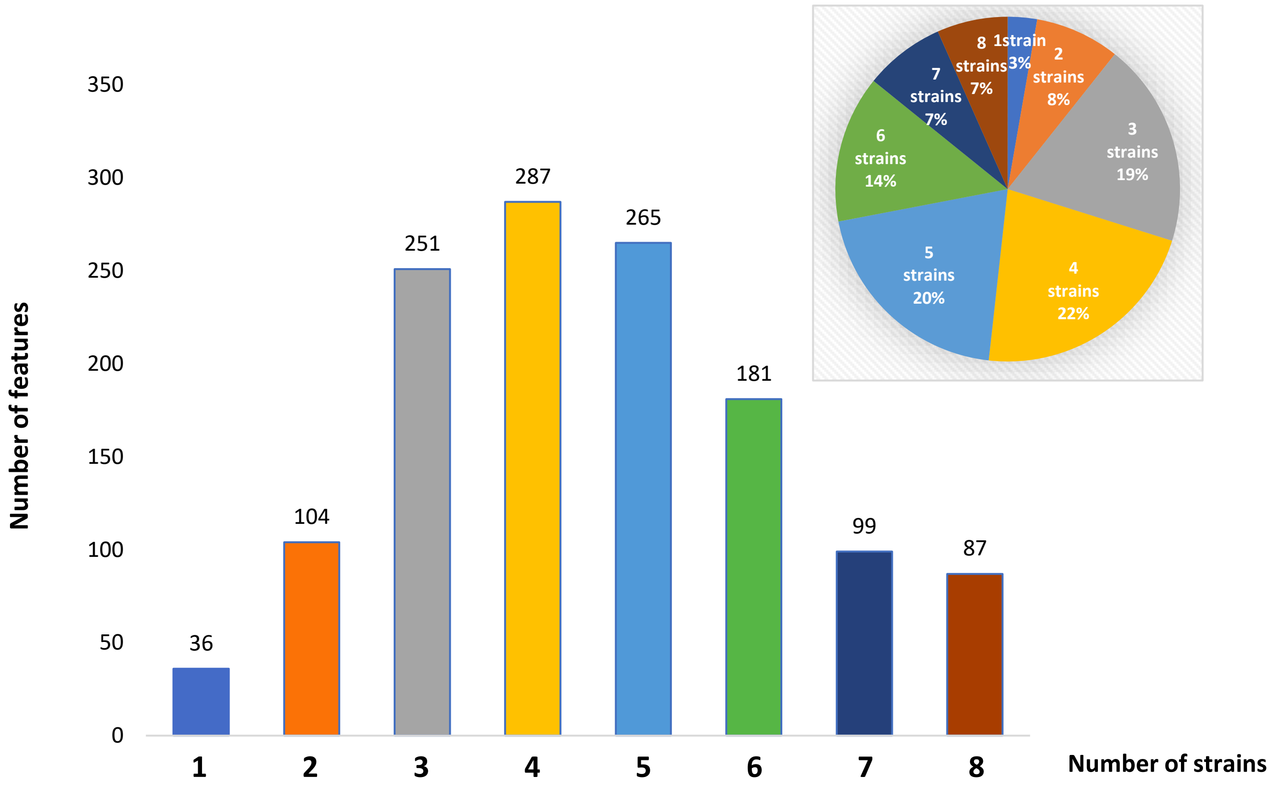

Fig. S4. Distribution of GS/MS features in strains.

Table S1. Bioassay evaluation of growth inhibition by *Streptomyces*.

		Receiver strain											
		Streptomyces								*Paenisporosarcina*	*Rhodococcus*		*Bacillus*
		RPA4-5	S1D4-11	RPA4-2	RLB1-33	RLB1-9	RLA2-12	RLB1-8	S1D4-23	RB2.1-N3	RB2.2-N6	RB2.1-N6	RB2.2-N4
Tester strain	RPA4-5												
	S1D4-11												
	RPA4-2												
	RLB1-33												
	RLB1-9												
	S1D4-23												
	RLA2-12												
	RLB1-8												
